# Evodiamide

**DOI:** 10.1107/S1600536811055553

**Published:** 2012-01-07

**Authors:** Fang Cai, Jie Wu, Hai-Yan Tian, Xiao-Feng Yuan, Ren-Wang Jiang

**Affiliations:** a Guangdong Province Key Laboratory of Pharmacodynamic Constituents of Traditional Chinese Medicine and New Drugs Research, Institute of Traditional Chinese Medicine and Natural Products, Jinan University, Guangzhou 510632, People’s Republic of China

## Abstract

The title compound, C_19_H_21_N_3_O, was isolated from the fruits of *Evodia rutaecarpa*. The indole and benzene rings are both essentially planar with mean derivations of 0.0094 (4) Å and 0.0077 (3) Å, respectively. The dihedral angle between these two planes is 78.24 (9)°. The amide carbonyl plane is roughly parallel to the indole ring with a dihedral angle of 7.0 (2)°, but makes a dihedral angle of 82.9 (3)° with the benzene ring. Inter­molecular N—H⋯O hydrogen-bonding inter­actions involving the amino and carbonyl groups give rise to a three-dimensional network.

## Related literature

For previous isolation of evodiamide, see: Shoji *et al.* (1988 ▶); Tang *et al.* (1997 ▶); Zuo *et al.* (2003 ▶). For the LC–MS analysis, see: Zhou *et al.* (2006 ▶). For the crystal structure of evodiamine, see: Fujii *et al.* (2000 ▶). For the biological activity of *Evodia rutaecarpa* and related alkaloids, see: Liao *et al.* (2011 ▶).
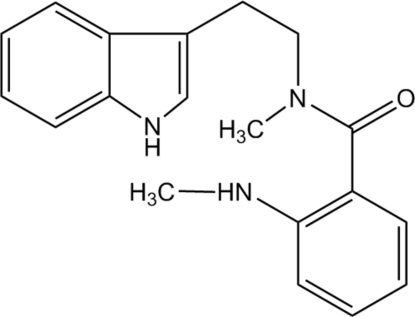


## Experimental

### 

#### Crystal data

C_19_H_21_N_3_O
*M* *_r_* = 307.39
Triclinic, 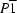

*a* = 8.958 (2) Å
*b* = 9.886 (2) Å
*c* = 10.616 (2) Å
α = 84.076 (4)°
β = 76.276 (4)°
γ = 66.746 (3)°
*V* = 839.0 (3) Å^3^
*Z* = 2
Mo *K*α radiation
μ = 0.08 mm^−1^
*T* = 291 K
0.45 × 0.36 × 0.30 mm

#### Data collection

Bruker SMART CCD 1000 diffractometer
Absorption correction: multi-scan (*SADABS*; Sheldrick, 2004 ▶) *T*_min_ = 0.661, *T*_max_ = 1.000
4931 measured reflections
3424 independent reflections
2149 reflections with *I* > 2σ(*I*)
*R*_int_ = 0.017

#### Refinement

*R*[*F* ^2^ > 2σ(*F* ^2^)] = 0.049
*wR*(*F* ^2^) = 0.146
*S* = 1.04
3424 reflections
211 parameters
H-atom parameters constrained
Δρ_max_ = 0.35 e Å^−3^
Δρ_min_ = −0.22 e Å^−3^

### 

Data collection: *SMART* (Bruker, 1998 ▶); cell refinement: *SMART* and *SAINT* (Bruker, 1998 ▶); data reduction: *XPREP* in *SHELXTL* (Sheldrick, 2008 ▶); program(s) used to solve structure: *SHELXTL*; program(s) used to refine structure: *SHELXTL*; molecular graphics: *XP* in *SHELXTL*; software used to prepare material for publication: *SHELXTL*.

## Supplementary Material

Crystal structure: contains datablock(s) I, global. DOI: 10.1107/S1600536811055553/vm2145sup1.cif

Structure factors: contains datablock(s) I. DOI: 10.1107/S1600536811055553/vm2145Isup2.hkl

Supplementary material file. DOI: 10.1107/S1600536811055553/vm2145Isup3.cml

Additional supplementary materials:  crystallographic information; 3D view; checkCIF report

## Figures and Tables

**Table 1:** Hydrogen-bond geometry (Å, °)

*D*—H⋯*A*	*D*—H	H⋯*A*	*D*⋯*A*	*D*—H⋯*A*
N1—H1*A*⋯O1^i^	0.86	1.99	2.824 (3)	164
N3—H3*A*⋯O1^ii^	0.86	2.39	2.991 (4)	128

Symmetry codes: (i) 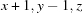; (ii) 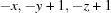.

**Fig. 1.**
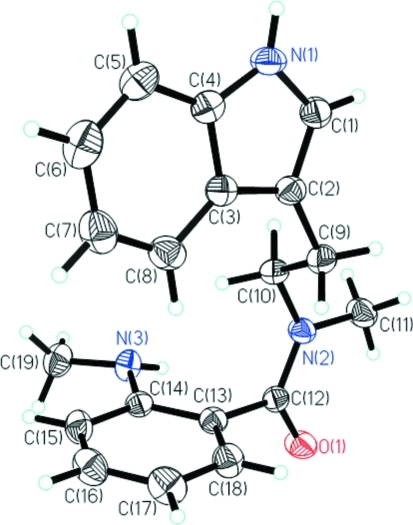
The molecular structure of the title compound showing 30% probability displacement ellipsoids.

**Fig. 2.**
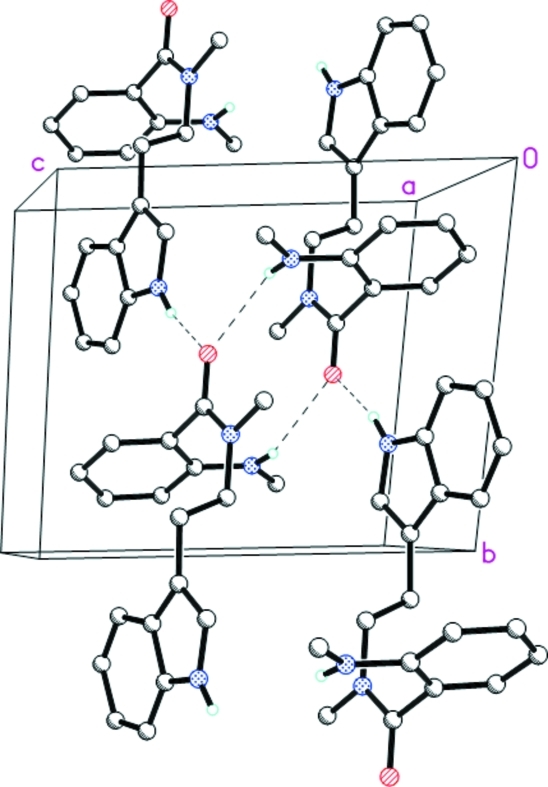
The packing diagram viewed down the a axis. The dashed lines represent intermolecular N—H···O hydrogen bonds. Selected H-atoms highlighting the hydrogen bonding are shown.

## supplementary crystallographic information

### Comment

The title compound was isolated from Evodia rutaecarpa which was purchased in Japan three decades ago and its structure was determined by chemical and spectral means (Shoji *et al.*, 1988). Since then, it was also isolated from the same herb collected in different locations of China (Tang *et al.*, 1997; Zuo *et al.*, 2003). Rapid detection of evodiamide and related alkaloids by LC–MS was reported (Zhou *et al.*, 2006). It was considered a precursor of evodiamine whose crystal structure was reported (Fujii *et al.*, 2000); however, the crystal structure of the title compound was not reported yet.

During the course of investigation of the bioactive compounds from Traditional Chinese Medicine, the title compound was isolated from Evodia rutaecarpa collected in Guangdong province of China. The colorless prisms of crystals were obtained from a methanol solution. It contains an indole ring, an amide functional group and a benzene ring (Fig. 1). Both the indole and benzene rings are planar with a mean derivation of 0.0094 (4) Å and 0.0077 (3) Å, respectively. The dihedral angle between these two planes is 78.24 (9)°. The amide carbonyl plane is roughly parallel to the indole ring with a dihedral angle of 7.0 (2)°, but makes a dihedral angle of 82.9 (3)° with the benzene ring C13-C18. Intermolecular N—H···O hydrogen-bonding interactions (Table 1) involving the amine and carbonyl groups give a three-dimensional network (Fig. 2).

### Experimental

Dried fruits (2.3 kg) of Evodia rutaecarpa (A. juss.) Benth. were milled and extracted with alcohol under reflux condition for 3 h. The alcohol extracts were filtered and concentrated to a syrup, which was suspended with water and sequentially partitioned with petroleum ether and ethyl acetate. The ethyl acetate extract (206 g) was chromatographied on silica gel with gradient elution dichloromethane-methanol to give 30 fractions. Fraction 8 eluted by dichloromethane-methanol (19:1) was further chromatographied on silica gel with chloroform-acetone (4:1) as the mobile phase to give title compound (15 mg). Colorless crystals were obtained by recrystallization from a methanol solution.

### Refinement

The C-bound H atoms were positioned geometrically and were included in the refinement in the riding-model approximation, with C—H = 0.96 Å (CH_3_) and *U*_iso_(H) = 1.5*U*_eq_(C); 0.97 Å (CH_2_) and *U*_iso_(H) = 1.2*U*_eq_(C); 0.93 Å (aryl H) and *U*_iso_(H)= 1.2*U*_eq_(C); O—H = 0.82 Å and *U*_iso_(H) = 1.5*U*_eq_(O).

### Crystal data

**Table:**

C_19_H_21_N_3_O	*Z* = 2
*M**_r_* = 307.39	*F*(000) = 328
Triclinic, *P*1	*D*_x_ = 1.217 Mg m^−^^3^
Hall symbol: -P 1	Mo *K*α radiation, λ = 0.71073 Å
*a* = 8.958 (2) Å	Cell parameters from 4931 reflections
*b* = 9.886 (2) Å	θ = 2.2–26.5°
*c* = 10.616 (2) Å	µ = 0.08 mm^−^^1^
α = 84.076 (4)°	*T* = 291 K
β = 76.276 (4)°	Prism, colorless
γ = 66.746 (3)°	0.45 × 0.36 × 0.30 mm
*V* = 839.0 (3) Å^3^	

### Data collection

**Table:**

Bruker SMART CCD 1000 diffractometer	3424 independent reflections
Radiation source: fine-focus sealed tube	2149 reflections with *I* > 2σ(*I*)
graphite	*R*_int_ = 0.017
ω scan	θ_max_ = 26.5°, θ_min_ = 2.2°
Absorption correction: multi-scan (*SADABS*; Sheldrick, 2004)	*h* = −10→11
*T*_min_ = 0.661, *T*_max_ = 1.000	*k* = −10→12
4931 measured reflections	*l* = −13→9

### Refinement

**Table:**

Refinement on *F*^2^	Primary atom site location: structure-invariant direct methods
Least-squares matrix: full	Secondary atom site location: difference Fourier map
*R*[*F*^2^ > 2σ(*F*^2^)] = 0.049	Hydrogen site location: inferred from neighbouring sites
*wR*(*F*^2^) = 0.146	H-atom parameters constrained
*S* = 1.04	*w* = 1/[σ^2^(*F*_o_^2^) + (0.0649*P*)^2^ + 0.1581*P*] where *P* = (*F*_o_^2^ + 2*F*_c_^2^)/3
3424 reflections	(Δ/σ)_max_ < 0.001
211 parameters	Δρ_max_ = 0.35 e Å^−^^3^
0 restraints	Δρ_min_ = −0.22 e Å^−^^3^

### Special details

**Table:**

Geometry. All e.s.d.'s (except the e.s.d. in the dihedral angle between two l.s. planes) are estimated using the full covariance matrix. The cell e.s.d.'s are taken into account individually in the estimation of e.s.d.'s in distances, angles and torsion angles; correlations between e.s.d.'s in cell parameters are only used when they are defined by crystal symmetry. An approximate (isotropic) treatment of cell e.s.d.'s is used for estimating e.s.d.'s involving l.s. planes.
Refinement. Refinement of *F*^2^ against ALL reflections. The weighted *R*-factor *wR* and goodness of fit *S* are based on *F*^2^, conventional *R*-factors *R* are based on *F*, with *F* set to zero for negative *F*^2^. The threshold expression of *F*^2^ > σ(*F*^2^) is used only for calculating *R*-factors(gt) *etc*. and is not relevant to the choice of reflections for refinement. *R*-factors based on *F*^2^ are statistically about twice as large as those based on *F*, and *R*- factors based on ALL data will be even larger.

### Fractional atomic coordinates and isotropic or equivalent isotropic displacement parameters (Å^2^)

**Table:**

	*x*	*y*	*z*	*U*_iso_*/*U*_eq_	
N1	0.7904 (2)	−0.28425 (19)	0.26473 (18)	0.0509 (5)	
H1A	0.8747	−0.3553	0.2853	0.069 (8)*	
N2	0.2948 (2)	0.31488 (17)	0.37369 (17)	0.0437 (4)	
N3	−0.0454 (2)	0.23860 (19)	0.48132 (17)	0.0485 (5)	
H3A	0.0126	0.2686	0.5169	0.135 (14)*	
O1	0.08389 (18)	0.52561 (15)	0.34402 (16)	0.0558 (4)	
C1	0.7523 (3)	−0.1380 (2)	0.2787 (2)	0.0490 (5)	
H1	0.8143	−0.1003	0.3127	0.059*	
C2	0.6114 (2)	−0.0550 (2)	0.2362 (2)	0.0438 (5)	
C3	0.5579 (2)	−0.1564 (2)	0.19235 (18)	0.0431 (5)	
C4	0.6720 (3)	−0.2988 (2)	0.21239 (19)	0.0459 (5)	
C5	0.6557 (3)	−0.4248 (3)	0.1821 (2)	0.0579 (6)	
H5	0.7322	−0.5180	0.1966	0.070*	
C6	0.5236 (4)	−0.4069 (3)	0.1305 (2)	0.0703 (7)	
H6	0.5089	−0.4895	0.1104	0.084*	
C7	0.4092 (3)	−0.2662 (3)	0.1069 (2)	0.0686 (7)	
H7	0.3213	−0.2574	0.0701	0.082*	
C8	0.4249 (3)	−0.1416 (3)	0.1372 (2)	0.0570 (6)	
H8	0.3485	−0.0488	0.1213	0.068*	
C9	0.5227 (3)	0.1091 (2)	0.2416 (2)	0.0494 (5)	
H9A	0.4777	0.1441	0.1644	0.059*	
H9B	0.6012	0.1537	0.2429	0.059*	
C10	0.3823 (2)	0.1555 (2)	0.3612 (2)	0.0452 (5)	
H10A	0.3030	0.1124	0.3580	0.054*	
H10B	0.4276	0.1165	0.4377	0.054*	
C11	0.3714 (3)	0.3946 (2)	0.4276 (2)	0.0586 (6)	
H11A	0.3025	0.4977	0.4307	0.088*	
H11B	0.4791	0.3800	0.3740	0.088*	
H11C	0.3831	0.3585	0.5137	0.088*	
C12	0.1543 (2)	0.3898 (2)	0.33301 (19)	0.0396 (5)	
C13	0.0829 (2)	0.3066 (2)	0.27095 (19)	0.0405 (5)	
C14	−0.0184 (2)	0.2372 (2)	0.34723 (19)	0.0405 (5)	
C15	−0.0836 (3)	0.1641 (2)	0.2837 (2)	0.0500 (6)	
H15	−0.1513	0.1177	0.3321	0.060*	
C16	−0.0495 (3)	0.1595 (3)	0.1507 (2)	0.0603 (6)	
H16	−0.0928	0.1085	0.1105	0.072*	
C17	0.0476 (3)	0.2289 (3)	0.0761 (2)	0.0629 (7)	
H17	0.0689	0.2268	−0.0140	0.076*	
C18	0.1131 (3)	0.3022 (2)	0.1380 (2)	0.0530 (6)	
H18	0.1791	0.3495	0.0885	0.064*	
C19	−0.1698 (3)	0.1902 (2)	0.5620 (2)	0.0609 (6)	
H19A	−0.2751	0.2438	0.5386	0.091*	
H19B	−0.1791	0.2077	0.6514	0.091*	
H19C	−0.1379	0.0869	0.5497	0.091*	

### Atomic displacement parameters (Å^2^)

**Table:**

	*U*^11^	*U*^22^	*U*^33^	*U*^12^	*U*^13^	*U*^23^
N1	0.0455 (10)	0.0397 (10)	0.0620 (12)	−0.0070 (8)	−0.0185 (9)	0.0013 (8)
N2	0.0400 (9)	0.0362 (9)	0.0556 (11)	−0.0119 (8)	−0.0160 (8)	−0.0015 (8)
N3	0.0515 (11)	0.0527 (11)	0.0452 (11)	−0.0260 (9)	−0.0070 (9)	0.0000 (8)
O1	0.0516 (9)	0.0349 (8)	0.0805 (11)	−0.0076 (7)	−0.0265 (8)	−0.0077 (7)
C1	0.0446 (12)	0.0448 (13)	0.0562 (14)	−0.0154 (10)	−0.0101 (10)	−0.0029 (10)
C2	0.0380 (11)	0.0406 (11)	0.0451 (12)	−0.0100 (9)	−0.0035 (9)	−0.0006 (9)
C3	0.0413 (11)	0.0475 (12)	0.0349 (11)	−0.0142 (10)	−0.0030 (9)	−0.0002 (9)
C4	0.0501 (12)	0.0459 (12)	0.0375 (11)	−0.0165 (10)	−0.0048 (9)	−0.0015 (9)
C5	0.0734 (17)	0.0495 (14)	0.0505 (14)	−0.0234 (12)	−0.0122 (12)	−0.0025 (10)
C6	0.100 (2)	0.0733 (18)	0.0512 (15)	−0.0477 (17)	−0.0130 (15)	−0.0067 (13)
C7	0.0757 (18)	0.095 (2)	0.0520 (15)	−0.0456 (17)	−0.0221 (13)	−0.0012 (14)
C8	0.0538 (14)	0.0681 (16)	0.0477 (13)	−0.0217 (12)	−0.0136 (11)	0.0051 (11)
C9	0.0430 (12)	0.0409 (12)	0.0588 (14)	−0.0131 (9)	−0.0083 (10)	0.0043 (10)
C10	0.0406 (11)	0.0371 (11)	0.0534 (13)	−0.0097 (9)	−0.0128 (10)	0.0043 (9)
C11	0.0540 (14)	0.0532 (14)	0.0775 (17)	−0.0210 (11)	−0.0279 (12)	−0.0047 (12)
C12	0.0385 (11)	0.0364 (11)	0.0428 (12)	−0.0135 (9)	−0.0083 (9)	−0.0009 (9)
C13	0.0375 (11)	0.0355 (11)	0.0448 (12)	−0.0097 (9)	−0.0076 (9)	−0.0049 (9)
C14	0.0381 (11)	0.0312 (10)	0.0483 (12)	−0.0086 (9)	−0.0093 (9)	−0.0028 (9)
C15	0.0497 (13)	0.0454 (12)	0.0596 (15)	−0.0228 (10)	−0.0095 (11)	−0.0078 (10)
C16	0.0615 (15)	0.0617 (15)	0.0640 (16)	−0.0237 (13)	−0.0178 (13)	−0.0171 (12)
C17	0.0641 (16)	0.0791 (17)	0.0457 (14)	−0.0256 (14)	−0.0105 (12)	−0.0124 (12)
C18	0.0523 (13)	0.0624 (14)	0.0456 (13)	−0.0253 (12)	−0.0060 (10)	−0.0029 (11)
C19	0.0628 (15)	0.0583 (15)	0.0599 (15)	−0.0287 (12)	−0.0008 (12)	0.0001 (11)

### Geometric parameters (Å, °)

**Table:**

N1—C1	1.363 (3)	C8—H8	0.9300
N1—C4	1.368 (3)	C9—C10	1.521 (3)
N1—H1A	0.8600	C9—H9A	0.9700
N2—C12	1.332 (2)	C9—H9B	0.9700
N2—C11	1.458 (3)	C10—H10A	0.9700
N2—C10	1.461 (2)	C10—H10B	0.9700
N3—C14	1.387 (3)	C11—H11A	0.9600
N3—C19	1.445 (3)	C11—H11B	0.9600
N3—H3A	0.8600	C11—H11C	0.9600
O1—C12	1.242 (2)	C12—C13	1.500 (3)
C1—C2	1.359 (3)	C13—C18	1.374 (3)
C1—H1	0.9300	C13—C14	1.406 (3)
C2—C3	1.425 (3)	C14—C15	1.392 (3)
C2—C9	1.499 (3)	C15—C16	1.373 (3)
C3—C8	1.400 (3)	C15—H15	0.9300
C3—C4	1.406 (3)	C16—C17	1.374 (3)
C4—C5	1.386 (3)	C16—H16	0.9300
C5—C6	1.362 (3)	C17—C18	1.385 (3)
C5—H5	0.9300	C17—H17	0.9300
C6—C7	1.404 (4)	C18—H18	0.9300
C6—H6	0.9300	C19—H19A	0.9600
C7—C8	1.372 (3)	C19—H19B	0.9600
C7—H7	0.9300	C19—H19C	0.9600
			
C1—N1—C4	108.52 (18)	N2—C10—C9	113.61 (17)
C1—N1—H1A	125.7	N2—C10—H10A	108.8
C4—N1—H1A	125.7	C9—C10—H10A	108.8
C12—N2—C11	119.29 (17)	N2—C10—H10B	108.8
C12—N2—C10	123.18 (17)	C9—C10—H10B	108.8
C11—N2—C10	117.46 (16)	H10A—C10—H10B	107.7
C14—N3—C19	120.82 (18)	N2—C11—H11A	109.5
C14—N3—H3A	119.6	N2—C11—H11B	109.5
C19—N3—H3A	119.6	H11A—C11—H11B	109.5
C2—C1—N1	110.75 (19)	N2—C11—H11C	109.5
C2—C1—H1	124.6	H11A—C11—H11C	109.5
N1—C1—H1	124.6	H11B—C11—H11C	109.5
C1—C2—C3	106.02 (18)	O1—C12—N2	121.55 (18)
C1—C2—C9	127.5 (2)	O1—C12—C13	120.06 (17)
C3—C2—C9	126.38 (19)	N2—C12—C13	118.39 (17)
C8—C3—C4	118.6 (2)	C18—C13—C14	119.92 (19)
C8—C3—C2	134.2 (2)	C18—C13—C12	119.34 (18)
C4—C3—C2	107.22 (18)	C14—C13—C12	120.70 (17)
N1—C4—C5	130.0 (2)	N3—C14—C15	122.18 (19)
N1—C4—C3	107.49 (18)	N3—C14—C13	119.84 (18)
C5—C4—C3	122.5 (2)	C15—C14—C13	117.93 (19)
C6—C5—C4	117.5 (2)	C16—C15—C14	121.0 (2)
C6—C5—H5	121.2	C16—C15—H15	119.5
C4—C5—H5	121.2	C14—C15—H15	119.5
C5—C6—C7	121.4 (2)	C15—C16—C17	121.1 (2)
C5—C6—H6	119.3	C15—C16—H16	119.4
C7—C6—H6	119.3	C17—C16—H16	119.4
C8—C7—C6	121.1 (2)	C16—C17—C18	118.4 (2)
C8—C7—H7	119.4	C16—C17—H17	120.8
C6—C7—H7	119.4	C18—C17—H17	120.8
C7—C8—C3	118.9 (2)	C13—C18—C17	121.6 (2)
C7—C8—H8	120.6	C13—C18—H18	119.2
C3—C8—H8	120.6	C17—C18—H18	119.2
C2—C9—C10	111.22 (17)	N3—C19—H19A	109.5
C2—C9—H9A	109.4	N3—C19—H19B	109.5
C10—C9—H9A	109.4	H19A—C19—H19B	109.5
C2—C9—H9B	109.4	N3—C19—H19C	109.5
C10—C9—H9B	109.4	H19A—C19—H19C	109.5
H9A—C9—H9B	108.0	H19B—C19—H19C	109.5
			
C4—N1—C1—C2	−0.3 (2)	C11—N2—C10—C9	−80.9 (2)
N1—C1—C2—C3	−0.1 (2)	C2—C9—C10—N2	178.10 (17)
N1—C1—C2—C9	176.33 (19)	C11—N2—C12—O1	−2.5 (3)
C1—C2—C3—C8	−179.1 (2)	C10—N2—C12—O1	−179.33 (18)
C9—C2—C3—C8	4.4 (4)	C11—N2—C12—C13	176.58 (18)
C1—C2—C3—C4	0.5 (2)	C10—N2—C12—C13	−0.2 (3)
C9—C2—C3—C4	−176.03 (19)	O1—C12—C13—C18	81.0 (2)
C1—N1—C4—C5	−179.0 (2)	N2—C12—C13—C18	−98.1 (2)
C1—N1—C4—C3	0.6 (2)	O1—C12—C13—C14	−97.0 (2)
C8—C3—C4—N1	179.03 (18)	N2—C12—C13—C14	83.9 (2)
C2—C3—C4—N1	−0.7 (2)	C19—N3—C14—C15	−12.8 (3)
C8—C3—C4—C5	−1.4 (3)	C19—N3—C14—C13	169.64 (18)
C2—C3—C4—C5	178.93 (19)	C18—C13—C14—N3	178.39 (18)
N1—C4—C5—C6	179.8 (2)	C12—C13—C14—N3	−3.6 (3)
C3—C4—C5—C6	0.4 (3)	C18—C13—C14—C15	0.8 (3)
C4—C5—C6—C7	0.9 (4)	C12—C13—C14—C15	178.76 (17)
C5—C6—C7—C8	−1.1 (4)	N3—C14—C15—C16	−177.4 (2)
C6—C7—C8—C3	0.1 (3)	C13—C14—C15—C16	0.2 (3)
C4—C3—C8—C7	1.1 (3)	C14—C15—C16—C17	−1.1 (4)
C2—C3—C8—C7	−179.3 (2)	C15—C16—C17—C18	1.1 (4)
C1—C2—C9—C10	−95.3 (3)	C14—C13—C18—C17	−0.9 (3)
C3—C2—C9—C10	80.5 (3)	C12—C13—C18—C17	−178.9 (2)
C12—N2—C10—C9	95.9 (2)	C16—C17—C18—C13	−0.1 (4)

### Hydrogen-bond geometry (Å, °)

**Table:**

*D*—H···*A*	*D*—H	H···*A*	*D*···*A*	*D*—H···*A*
N1—H1A···O1^i^	0.86	1.99	2.824 (3)	164
N3—H3A···O1^ii^	0.86	2.38	2.991 (4)	128

Symmetry codes: (i) *x*+1, *y*−1, *z*; (ii) −*x*, −*y*+1, −*z*+1.
